# White matter volume loss drives cortical reshaping after thalamic infarcts

**DOI:** 10.1016/j.nicl.2022.102953

**Published:** 2022-02-04

**Authors:** Julian Conrad, Maximilian Habs, Ria M. Ruehl, Rainer Bögle, Matthias Ertl, Valerie Kirsch, Ozan E Eren, Sandra Becker-Bense, Thomas Stephan, Frank A Wollenweber, Marco Duering, Peter zu Eulenburg, Marianne Dieterich

**Affiliations:** a Department of Neurology, University Hospital, LMU Munich, Germany; b German Centre for Vertigo and Balance Disorders (DSGZ), University Hospital, LMU Munich, Germany; c Munich Centre of Neuroscience, Munich, Germany; d Department of Psychology, University of Bern, Switzerland; e Institute for Stroke and Dementia Research (ISD), University Hospital, LMU Munich, Germany; f Department of Neurology, Helios Dr. Horst Schmidt Kliniken, Wiesbaden, Germany; g Institute for Neuroradiology, University Hospital, LMU Munich, Germany; h Munich Cluster for Systems Neurology (SyNergy), Munich, Germany

**Keywords:** Thalamus, Vbm, Vestibular, Ocular motor, Stroke

## Abstract

**Objective:**

The integration of somatosensory, ocular motor and vestibular signals is necessary for self-location in space and goal-directed action. We aimed to detect remote changes in the cerebral cortex after thalamic infarcts to reveal the thalamo-cortical connections necessary for multisensory processing and ocular motor control.

**Methods:**

Thirteen patients with unilateral ischemic thalamic infarcts presenting with vestibular, somatosensory, and ocular motor symptoms were examined longitudinally in the acute phase and after six months. Voxel- and surface-based morphometry were used to detect changes in vestibular and multisensory cortical areas and known hubs of central ocular motor processing. The results were compared with functional connectivity data in 50 healthy volunteers.

**Results:**

Patients with paramedian infarcts showed impaired saccades and vestibular perception, i.e., tilts of the subjective visual vertical (SVV). The most common complaint in these patients was double vision or vertigo / dizziness. Posterolateral thalamic infarcts led to tilts of the SVV and somatosensory deficits without vertigo. Tilts of the SVV were higher in paramedian compared to posterolateral infarcts (median 11.2° vs 3.8°). Vestibular and ocular motor symptoms recovered within six months. Somatosensory deficits persisted. Structural longitudinal imaging showed significant volume reduction in subcortical structures connected to the infarcted thalamic nuclei (vestibular nuclei region, dentate nucleus region, trigeminal root entry zone, medial lemniscus, superior colliculi). Volume loss was evident in connections to the frontal, parietal and cingulate lobes. Changes were larger in the ipsilesional hemisphere but were also detected in homotopical regions contralesionally. The white matter volume reduction led to deformation of the cortical projection zones of the infarcted nuclei.

**Conclusions:**

White matter volume loss after thalamic infarcts reflects sensory input from the brainstem as well the cortical projections of the main affected nuclei for sensory and ocular motor processing. Changes in the cortical geometry seem not to reflect gray matter atrophy but rather reshaping of the cortical surface due to the underlying white matter atrophy.

## Highlights

• White matter volume loss after unilateral thalamic infarcts shows the trajectories of sensory and ocular motor input from the brainstem to the thalamus and their thalamocortical connections.
• The extensive volume loss drives reshaping of the cortex more than grey matter atrophy. Associated ocular motor and vestibular symptoms are compensated over time due to their redundant and intermingled connectivity and an early integration with other sensory modalities.
• Associated ocular motor and vestibular symptoms are compensated over time due to their redundant and intermingled connectivity and an early integration with other sensory modalities.

## 1 Introduction

Due to its location and widely distributed connectivity, the thalamus plays an integral role in most cortical functions. Thalamocortical connectivity can be considered a key to decipher the neural basis of human behavior. (Halassa and Sherman, 2019) Symptoms of thalamic infarcts range from impaired visual and somatosensory perception to higher order multisensory and cognitive disorders. The clinical syndrome depends on the territorial vascular anatomy and the thalamic subnuclei that are affected by unilateral or bilateral infarcts. (Li et al., 2018)

Vestibular and ocular motor processing has been identified in numerous thalamic subnuclei. (Wijesinghe et al., 2015, Sommer and Wurtz, 2004) The mediodorsal thalamic nucleus (MD), for example, connects - among other functions - the superior colliculus (SC) with the premotor cortex including the frontal eye fields (FEF) for saccade processing. (Sommer and Wurtz, 2004) Posterior and lateral thalamic nuclei neurons respond to multiple sensory stimuli. (Wijesinghe et al., 2015, Meng and Angelaki, 2010, Dale and Cullen, 2019) In humans, data on thalamic vestibular processing is sparse. Lesion studies identified two centers that seem to convey vestibular perception of verticality from the thalamus. (Baier et al., 2016) An impaired activation of the ipsilateral parieto-opercular insular vestibular cortex was observed after caloric irrigation in patients with unilateral posterolateral thalamic infarcts. (Dieterich et al., 20052005) Recent imaging studies reported atrophy of the ipsilesional somatosensory thalamocortical tract and somatosensory cortex after posterolateral infarcts with changes in functional connectivity (fc) between thalamus, somatosensory and prefrontal cortex. (Chen et al., 2019, He et al., 2021)

In the current study, we aimed to extend our understanding of thalamocortical processing to longitudinal effects after acute unilateral thalamic infarcts on cortical sensory and ocular motor processing in stroke patients with somatosensory, vestibular, and ocular motor symptoms. Our hypothesis was that acute unilateral lesions induce processes of central compensation by reorganization of sensory-motor networks within the ipsilateral hemisphere and - probably to a lesser extent - the contralateral hemisphere that might differ for the sensory systems. We chose structural longitudinal imaging with voxel-based (*VBM*) and surface-based morphometry (*SBM*) because it allows a strict evaluation of intra-individual brain changes from the acute to the chronic phase without the possibility of introducing artefacts due to changes in hemodynamics and water diffusion in the acute and subacute phase. We asked whether (i) somatosensory, vestibular, and ocular motor symptoms following thalamic infarcts show a similar road to recovery, (ii) thalamic infarcts elicit structural reorganization in the cerebral cortex over time, (iii) compensation leads to volumetric changes in known cortical ocular motor, multisensory vestibular and somatosensory areas, and (iv) these reflect functional and structural connectivity profiles of the main affected nuclei.

## 2 Subjects and methods

### 2.1 Patients

We studied thirteen patients with isolated unilateral ischemic thalamic infarcts (eight females) who presented to our tertiary referral center (Department of Neurology, University Hospital, LMU, Munich, Germany) between 2012 and 2020 prospectively. Only patients that were able to complete the full radiological and clinical workup in the acute stage (M0) and after six months (M6) were included in the study (mean time to follow-up: 6.8 months (SD +/-1.3, range: 5–9 months)). Inclusion criteria were: Unilateral circumscribed ischemic thalamic infarct, confirmed by MR imaging, involving either the paramedian or posterolateral thalamus; ability to complete the detailed vestibular and ocular motor examination, and completion of follow-up imaging, vestibular and ocular motor examination after six months. Exclusion criteria were: Absence of an ischemic lesion on diffusion MRI, bilateral or multifocal infarcts, prior stroke, tumor, cerebral hemorrhage, vascular malformation, edema (i.e., compression of CSF space, shift of midline structures), severe white matter hyperintensities (WMH, Fazekas grade >1 for periventricular WMH and deep WMH) and if patients were unable to complete the neurological and neuro-ophthalmological examination due to cognitive impairment or impaired vigilance either in the acute phase or at the time of follow-up.

### 2.2 Healthy controls for functional connectivity analysis (fcHC)

State-of-the art resting-state functional connectivity (rsfcMRI) data with optimal temporal signal-to-noise ratio in the thalamus were acquired in a separate group of 50 healthy volunteers (fcHC; age range: 20.5–32.3 years, mean age: 24.4 ± 3.7 years, 25 female).

### 2.3 Clinical examination

A detailed neurological and neurotological examination was carried out in all patients at baseline and after six months. This included the neuroorthoptic examination of spontaneous eye position and eye movements (nystagmus), smooth pursuit, saccades, optokinetic nystagmus, vestibulo-ocular reflex (VOR), and measurements of the perception of verticality by the subjective visual vertical (SVV, for details see Table 1). For the SVV, a mean deviation of > ± 2.5° over seven measurements was considered pathological. (Baier et al., 2016) Presence of spatial neglect was evaluated using clinical examination (i.e., if patient failed to respond, orient or execute motor commands towards stimuli presented in the contralesional hemifield) and paper–pencil tests (Bells test, Albert’s test, line bisection). Contraversive pushing was diagnosed if patients showed lateropulsion to the contralateral hemisphere, actively pushed towards the side of the paresis and opposed efforts to correct the deviation of their body midline.

**Table 1:** Demographic and clinical data of the thirteen patients with thalamic infarcts.

**Demographic data**				
	**Patients**			

Age (median, range (y))	72 y (46–80 y; IQR 27)			
Handedness n (%) Right Left	13 (100) 0 (0.0)			
Gender n (%) Female Male	8 (61.5) 5 (38.5)			
**Clinical data**				
Infarct side n (%) Right Left	4 (30.8) 9 (69.2)			
Symptom onset to MRI (days, d; median, range, IQR)	2 d (1–3; IQR 2)			
Time to neuroorthoptic examination (days, d; mean, SD)	3.39 d (+/- 2.29)			
Time to follow-up (months, m; median, range, IQR)	6.7 m (4.5–9 m, IQR 1.5)			
	**M0**		**M6**	
**NIHSS score (median, range)**	2 (0–7)		0 (0–2)	
**Clinical test**	**n**	**%**	**n**	**%**

**Vestibular and ocular motor perceptual**				
Rotational vertigo	1/13	7.7	0/13	0.0
Dizziness	2/13	15.4	0/13	0.0
Double vision	5/13	38.5	1/13	7.7
**Vestibular**				
Lateropulsion	1/13	7.7	0/13	0.0
Falls	5/13	38.5	1/13	7.7
Pathological SVV score	6/13	46.2	0/13	0.0
SVV (mean °, SD)	4.2 (+/- 3.97)		1.04 (+/- 1.55)	
Skew deviation	3/13	23.1	0/13	0.0
Ocular torsion	3/13	23.1	0/13	0.0
Head tilt	4/13	30.8	0/13	0.0
Nystagmus (UBN, torsional)	2/13	15.4	0/13	0.0
Pathological VOR (head impulse test)	0/13	0.0	0/13	0.0
**Ocular motor**				
Saccades				
Slowing				
- All - Vertical - Horizontal	6/13 4/13 2/13	46.15 30.77 15.4	0/13 0/13 0/13	0.0 0.0 0.0
Hypometria				
- All - Horizontal - Vertical	3/13 2/13 3/13	23.1 15.4 23.1	0/13 0/13 0/13	0.0 0.0 0.0
Saccade palsy				
- Vertical	1/13	7.7	0/13	0.0
Post saccadic rotation (riMLF)	1/13	7.7	0/13	0.0
Dysmetria of saccades	4/13	30.8	2/13	15.4
Vertical saccade palsy	4/13	30.8	0/13	0.0
Impaired optokinetic reflex	3/13	23.1	0/13	0.0
Saccadic smooth pursuit	9/13	69.2	5/13	39.1
**Motor**				
Paresis	6/13	46.2	3/13	23.1
Ataxia	1/13	7.7	1/13	7.7
Dysarthria	3/13	23.1	0/13	0.0
**Somatosensory, visual and higher order multisensory**				
Hypesthesia	7/13	53.8	6/13	46.2
Visual field defect	1/13	7.7	0/13	0.0
Spatial neglect	1/13	7.7	0/13	0.0
Contraversive pushing	0/13	0.0	0/13	0.0
**Cognitive / Vigilance**				
Cognitive deficits	1/13	7.7	0/13	0.0
Impaired vigilance	1/13	7.7	0/13	0.0

SVV subjective visual vertical, UBN upbeating nystagmus, VOR vestibulo-ocular reflex, SD standard deviation, IQR interquartile range.

### 2.4 Imaging

All patients and HC underwent similar high resolution structural MR-imaging on a clinical 3T MRI scanner (T1 FSPGR, 1 mm^3^ isotropic, 176 slices, TR 6500 ms, TE 3.15 ms for GE Signa Excite HD (Milwaukee, WI, USA) or T1 MPRAGE, 1 mm^3^ isotropic, 192 slices, TR 2500 ms, TE 4.37 ms for Magnetom Verio or Magnetom Skyra (Siemens Healthcare, Erlangen, Germany)). Patients were evenly distributed over the MRI scanners (eight patients GE, five Siemens Verio or Skyra); the latter are part of the DEDEMAS study. (Wollenweber et al., 2014) All patients that were included had their follow-up MRIs on the same scanner.

#### 2.4.1 Lesion segmentation

Ischemic infarcts were all delineated on acute phase diffusion weighted imaging sequences (DWI, 3 mm TE = 880 ms TR = 1400 ms, 48 slices) using MRICRON (https://www.nitrc.org/projects/mricron). All lesion maps were then normalized into 1 × 1 × 1 mm^3^ MNI space using the *Clinical Toolbox* in SPM for visualization. (Rorden et al., 2012) The normalized lesions were compared with the high resolution T1 at follow-up for congruency. For the analysis, right-sided infarcts (n = 4) were flipped to the left.

#### 2.4.2 Voxel-based morphometry (VBM)

Data quality estimation, pre-processing, and analysis were performed using the longitudinal design option of the CAT12 toolbox, version 1739 (Gaser & Dahnke, Department of Psychiatry, University of Jena, Jena, Germany; http://www.neuro.uni-jena.de/cat) within Statistical Parametric Mapping SPM12, version 7771 (https://www.fil.ion.ucl.ac.uk/spm/; Wellcome Department of Cognitive Neurology), using Matlab R2019b (Mathworks) after standard preprocessing including a 6 mm Gaussian smoothing kernel. The modulated GM and WM images were used for the volumetric analysis. The sample homogeneity analysis revealed an excellent correlation of all signal characteristics within the sample.

#### 2.4.3 Surface-based analyses (SBM)

The CAT12 toolbox contains a fully implemented and validated processing pipeline for SBM. (Kharabian Masouleh et al., 20202020) We employed its established algorithm for extracting multiple geometric and structural parameters of the cortical surface. (Yotter et al., 2011, Dahnke et al., 2013)The morphometric parameters that can be computed are the cortical thickness (CT), the sulcus depth (SD), the fractional dimension (FD) and gyrification indices (GI) to analyze surface complexity based on the absolute mean curvature approach. (Luders et al., 2006) Therefore, the T1-weighted images underwent tissue segmentation to estimate white matter distance. Local maxima were then projected on to other gray matter voxels by using a neighbor relationship, described by the WM distance. These values equal cortical thickness. This projection-based method also includes partial volume correction, sulcal blurring and sulcal asymmetries without sulcus reconstruction. Topological correction was performed using an approach based on spherical harmonics. For inter-participant analysis, an algorithm for spherical mapping of the cortical surface was included. (Yotter et al., 2011, Dahnke et al., 2013) An adapted volume based diffeomorphic DARTEL algorithm was then applied to the surface for spherical registration. Central cortical surfaces were created for both hemispheres separately. Surface reconstructions of the cortical values for each hemisphere were resampled to the 164 k mesh template space (*Freesurfer*) after merging and then smoothed with a 14 mm (cortical thickness), 20 mm (sulcus depth) and 23 mm (fractal dimension and gyrification index) Gaussian filter, respectively.

#### 2.4.4 Resting-state functional connectivity (rsfc)

Two separate sessions (900 volumes each) of resting-state data were collected. The data were acquired on a 3T Magnetom Prisma scanner (Siemens Healthcare, Erlangen, Germany) using a 64-channel head/neck coil with eyes open using a custom 3D-EPI sequence (TR 570 ms, TE 33 ms, 2.4 mm isotropic voxel size). A functional connectivity analysis was performed with the rsfMRI data using CONN toolbox version 20b (http://www.nitrc.org/projects/) for Matlab (version 2019b). (Whitfield-Gabrieli and Nieto-Castanon, 2012) Movement parameters, quality control time series, and scrubbing regressors were employed by the CONN Toolbox as first level covariates. For denoising, the following nuisance regressor time series were used to lessen their impact: white matter (WM) and cerebrospinal fluid (CSF) confounds were each considered with their first five principal components. Furthermore, six principal temporal components of the movement parameters (three translation and three rotation parameters) were used. Images were denoised with a temporal band-pass filter (0.008–0.09 Hz).

### 2.5 Statistical analysis

For the VBM and SBM analysis, timepoints M0 and M6 of all patients were included in a longitudinal flexible factorial model in SPM12. Age and time to follow-up were used as covariates of no interest. To ensure the detection of all possible volumetric changes related to thalamic infarcts, right-sided infarcts (n = 4) were flipped to left for the analysis. T contrasts were estimated to detect differences between the groups. The results were corrected on the cluster level using non-parametric permutation testing (*threshold free cluster enhancement, TFCE*) as implemented in the CAT12 toolbox calculating up to 10,000 permutations. (Smith and Nichols, 2009) All results were corrected for multiple comparisons on the cluster level using *false discovery rate (FDR)* correction; p < 0.05. Imaging results are reported as being either ipsilesional or contralesional.

SBM results were displayed using the *Freesurfer* template with the multimodal Human connectome (HCP) parcellation implemented in the CAT12 toolbox (http://surfer.nmr.mgh.harvard.edu/). (Glasser et al., 2016) VBM and rsfc results were visualized using MRIcroGL (https://www.mccauslandcenter.sc.edu/mricrogl/).

For the fc-analysis, we calculated the overlap map of the lesions with the main affected nuclei, VPL and MD. In this analysis, only voxels that were affected in >4 patients and belonged to either of the two nuclei were used. The thresholded lesion maps were flipped to the contralateral hemisphere to facilitate the computation of whole brain connectivity from the seed regions. The seed-to-voxel results were thresholded at p < 0.001 FWE corrected (two-sided) together with 5000 permutations for non-parametric thresholding within the CONN toolbox. Results were shown with their peak t-score intensities.

### 2.6 Patient consent and data availability

The study was performed in accordance with the 1964 Declaration of Helsinki (latest applicable revision Fortaleza 2013) and approved by the institutional review board of LMU, Munich, Germany (no.094-10). All patients gave informed written consent to participate in the study. The rsfc data and lesion maps will be publicly available (https://osf.io/2nmcf/). The structural imaging patient data is not publicly available due to European Privacy laws and lack of consent for data sharing by the patients.

## 3 Results

### 3.1 Clinical

Left-sided infarcts were observed in nine patients, four had right-sided thalamic infarcts. Six infarcts were in the paramedian, and seven in the posterolateral thalamus. Pathological tilts of the SVV were present in six patients in the acute phase (two right-sided infarcts, four left-sided). Mean time to the SVV measurement was three days (3.38; SD ±2.29). The mean tilt of the SVV in all patients was ± 4.2° (SD ± 3.9°, range 0.6–11.2). Higher SVV tilts were observed in paramedian compared to posterolateral thalamic infarcts (median pathological SVV tilt in paramedian infarcts; 11.2°, range: 6.60°–11.50°; in posterolateral infarcts: 3.40°, range 3.20°–4.85°).

All patients with paramedian thalamic infarcts demonstrated components of ocular tilt reaction (OTR) due to an involvement of the paramedian ocular motor integration centers in the rostral midbrain (interstitial nucleus of Cajal, INC) accompanying paramedian thalamic infarcts. Double vision and vertigo (one rotational, four dizziness) were found in five of the six patients. Four of the six patients with paramedian infarcts displayed a saccade generation pathology (see Table 1 for details).

Sensory deficits were present in all seven patients with posterolateral infarcts. Four of these had hemihypesthesia; three reported reduced sensation of touch of the face and arm. Mild motor symptoms (mean severity of paresis according to Medical Research Council scoring was 3.9/5 (minimal motor strength was considered, range: 0–5)) were present in seven patients, ataxia in one, consistent with the clinical symptomatology of posterolateral thalamic infarcts. (Schmahmann, 2003) One patient demonstrated personal and peripersonal neglect. Here, the infarct extended towards the internal capsule and the lateral pulvinar. Falls were documented in five patients (four in posterolateral infarcts, one of these four with thalamic astasia).

We did not observe hearing disturbances and (contraversive) pusher syndrome in our patient group with isolated thalamic lesions. Dysarthria was reported in two patients with paramedian infarcts. Cognitive deficits were present in one patient with a paramedian infarct that extended towards the ventral anterior subnuclei. The deficit involved encoding new memories and phonologic more than semantic fluency. One patient with a paramedian infarct presented with a fluctuating impairment of vigilance.

The symptoms that led patients to the emergency room (i.e., the first symptom reported by the patients) were ocular motor or vestibular perceptual (double vision, vertigo / dizziness) deficits in paramedian infarcts and sensory deficits or hemiparesis in posterolateral infarcts. In the latter, perceptual correlates of the vestibular or ocular motor systems were not reported at all.

At follow-up, vestibular and ocular motor symptoms were fully compensated after six months (M6). In contrast, sensory and motor symptoms showed a poorer road to recovery. After six months, fortunately, half of the patients with paresis (n = 3) recovered. Sensory deficits were still present in six of seven patients with comparably little change in intensity, with two of them having developed a thalamic pain syndrome. Both patients had a thermoperception deficit at presentation.

### 3.2 Structural imaging

#### 3.2.1 Lesions

**Fig. 1:**
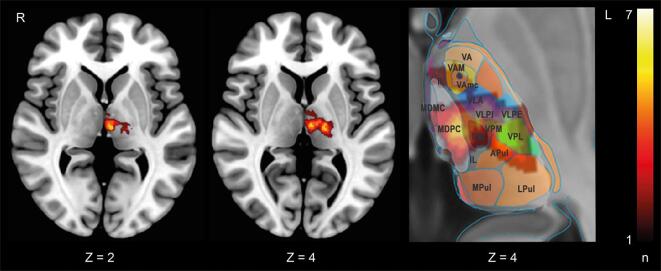
Overlap image of the thalamic infarcts. Lesion images were flipped to the left to create the overlap. Depiction of the thalamic nuclei adapted from Mai and Majtanik. (Mai and Majtanik, 2018) CM central medial nucleus (includes parafascicular nucleus), IL intralaminar nuclei, MD (MDMC, MDPC) mediodorsal nucleus, Pul (APul, DPul, LPul, MPul) anterior, medial, lateral pulvinar, VA (VA, VAM, VAmc, VAL) ventral anterior nucleus, VL (VLA, VLPI, VLPE) ventral lateral nucleus, VPL ventral posterior lateral nucleus, VPM ventral posterior medial nucleus. The color scale shows the number of lesions overlapping at a specific location.

Mean lesion volume was 1049.5 mm^3^ (range: 90–4990 mm^3^). The volume of paramedian infarcts was similar to that of posterolateral infarcts. The strongest overlap centered on the ventral posterior lateral (VPL) / ventral lateral nuclei (VL) and mediodorsal nucleus (MD). Infarcts extended into the central median nucleus (CM), the intralaminar nuclei including the parafascicular nucleus (Pf, included in CM in the atlas) and ventroposteromedial (VPM) nucleus. The paramedian and tuberothalamic arteries were the most commonly affected vessels^20^ (see Fig. 1; anatomical map adapted from Mai and Majtanik). (Mai and Majtanik, 2018)

#### 3.2.2 Surface based morphometry (SBM)

**Fig. 2:**
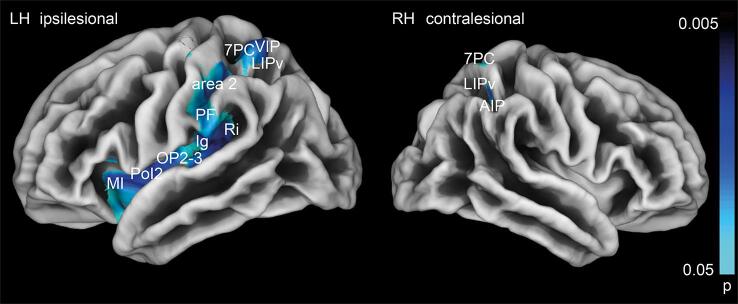
Changes in cortical geometry (gyrification index) at M6 compared to M0 are detected in area 2, the IPL (area PF), the insula (area Ri, Ig, PoI2, MI) and along the intraparietal sulcus (areas LIP / VIP, AIP) in the ipsilesional hemisphere and the superior parietal lobule (area 7PC) in both hemispheres. This likely reflects reshaping of the cortical architecture after white matter loss and not gray matter atrophy. Results are depicted with logarithmic p-value scales *FDR* corrected for *TFCE* after 10.000 permutations.

In the SBM analysis, we detected a decrease in cortical gyrification (gyrification index, GI) in area 2 of the postcentral gyrus extending to the intraparietal sulcus (cytoarchitectonic areas LIPv/d, VIP, AIP) and superior parietal lobule (7PC, 7AL) in the ipsilesional hemisphere. (Glasser et al., 2016) This decrease extended rostrally to the inferior parietal lobule (PF) and ventrally to the insula (areas Ri, Ig, OP2-3, PoI2, MI). In the contralesional hemisphere, we observed a reduction of GI in area 7PC/7AL and area AIP/LIPv (Fig. 2). Notably, we did not detect decreases in cortical thickness (CT) as a surrogate marker for cortical atrophy.

#### 3.2.3 Voxel-based morphometry (VBM)

In the VBM analysis, we did not detect clusters of gray matter volume changes after six months in the longitudinal analysis.

**Fig. 3:**
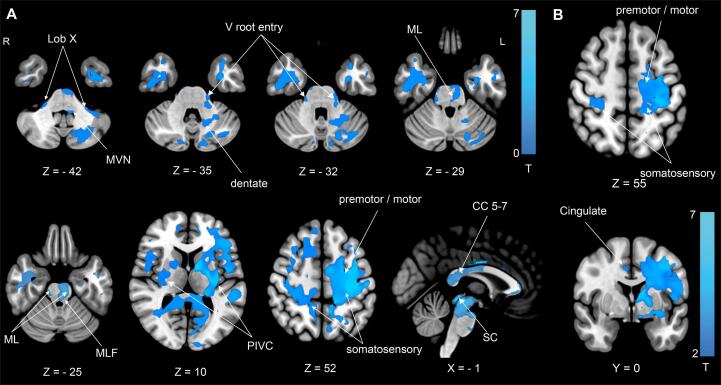
**(A)** WMV reduction at M6 compared to M0 demonstrates volume loss in the cerebellum (dentate nuclei region, lobule X), the medulla (middle vestibular nuclei, MVN), the pons (entry zone of the trigeminal nerve (V root entry zone), medial lemniscus (ML), medial longitudinal fascicle (MLF) and the midbrain including the superior colliculi (SC). In the cerebral hemispheres, WMV reduction is most prominent in the ipsilesional thalamocortical pathways connecting the thalamus with parietal opercular (retro-)insular cortex (PIVC), the (pre-) motor, somatosensory and cingulate cortex. Additional WMV decreases were located in ipsilesional and contralesional intrahemispheric association tracts and the corpus callosum (CC 5–7). The latter connects somatosensory, parietal cortex and PIVC of both hemispheres. **(B)** WMV reduction is most prominent in the thalamocortical projections with PIVC and (pre-) motor cortex in the ipsilesional hemisphere. WMV reduction in somatosensory and cingulate cortex is also found in the contralesional hemisphere. All results thresholded at p < 0.05, *FDR* corrected after calculating 10,000 permutations using *TFCE.*

We observed a profound reduction of white matter volume (WMV) ipsilesionally as well as contralesionally. The WMV loss included cerebellar and brainstem structures that are involved in ocular motor, vestibular and somatosensory processing and are connected to the infarcted thalamic subnuclei. Ipsilesional volume loss included the dentate nuclear region, the vestibular nuclear region (VN), the middle longitudinal fascicle (MLF), and the corticospinal tract (CST). As a general rule, WMV changes were larger in the ipsilesional hemisphere. The WMV reduction covered the mesencephalon, particularly the midline structures for ocular motor control (includes III, INC, riMLF, MLF). Additional contralesional WMV loss (i.e., bilateral volume loss) was seen in cerebellar lobule X (flocculus), the trigeminal root entry zone (V root entry zone), the medial lemniscus (ML) and the superior colliculi (SC; Fig. 3A).

In the cerebral hemispheres, we observed WMV loss ipsilesionally as well as contralesionally. Ipsilesional WMV loss was larger and T values were higher compared to contralesional WMV changes. WMV decreases were most prominent in the white matter connecting the thalamus with the parieto-opercular / (retro-) insular cortex (PIVC), (pre-) motor cortex in the ipsilesional and somatosensory and cingulate cortex in both hemispheres (Fig. 3B). The pattern of WMV reduction reflected cortical targets of the infarcted nuclei and their involvement in somatosensory processing and ocular motor control (Fig. 3B). WMV decreases were also observed in ipsilesional intrahemispheric association tracts that connect the temporoparietal with the premotor and rostral prefrontal cortex (mainly SLF). Further WMV reductions were present in the posterior part of the corpus callosum (CC5-7) connecting the somatosensory and vestibular cortices with the posterior parietal cortex of both hemispheres (Fig. 3A), as described in a functional connectivity study in healthy volunteers. (Kirsch et al., 2016) Contralesional volume decreases were also found in the thalamocortical projections centered around the PIVC, somatosensory and premotor cortex. Involvement of intrahemispheric association pathways was found to a lesser extent compared to the ipsilesional hemisphere and was more circumscribed around the cortical projections of the thalamic subnuclei.

### 3.3 Functional imaging

#### 3.3.1 Resting-state functional connectivity in the fcHC

**Fig. 4:**
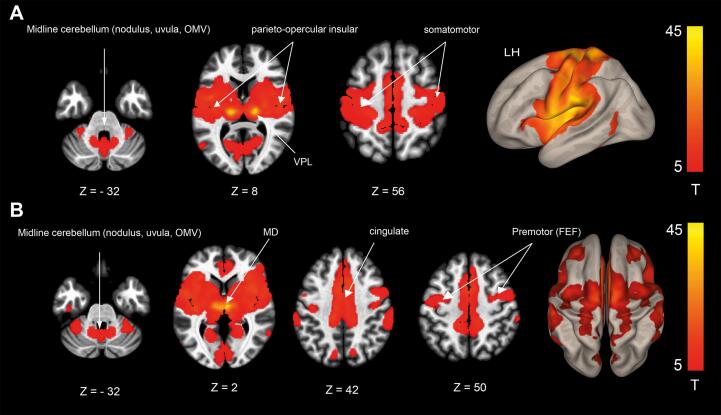
Resting-state functional connectivity (rsfc) analysis in healthy volunteers. **(A)** Rsfc of the VPL seed (overlap of thalamic infarcts with the ventral posterior lateral nucleus). Rsfc is demonstrated between the midline cerebellum, including key structures for ocular motor and vestibular processing such as the ocular motor vermis (OMV), nodulus and uvula, MD, the parieto-opercular insular (PIVC) and somatosensory cortex. (**B)** Rsfc of the MD seed (overlap of thalamic infarcts with the mediodorsal nucleus). Rsfc is demonstrated between the midline cerebellum, including key structures for ocular motor and vestibular processing such as the ocular motor vermis (OMV), nodulus and uvula, MD, the cingulate and premotor cortex including the frontal eye fields (FEF). Results thresholded at p < 0.001 *FWE* corrected after calculating 5000 permutations using *TFCE*.

Functional connectivity of the lesion-VPL seed was observed with the midline cerebellum (including the ocular motor vermis – OMV, nodulus and uvula and the dentate nuclei region) and the multisensory cortical areas (PIVC, somatosensory cortex and intraparietal sulcus), overlapping with the reduction of gyrification in the postcentral gyrus, the inferior parietal lobule (PF), insula and the WMV reductions around the PIVC (area OP2, Ig, Ri among others) in the patient sample (Fig. 4A).

Functional connectivity of the lesion-MD seed yielded connectivity of the midline cerebellum (OMV, nodulus, uvula) with the cingulum, premotor cortices (FEF) and the PIVC (Fig. 4B).

## 4 Discussion

The main findings can be summarized as follows: (i) Vestibular and ocular motor symptoms after thalamic infarcts were compensated within six months. Somatosensory symptoms had a lower rate of recovery. (ii) Thalamic infarcts led to a profound WMV reduction in the ipsilesional and contralesional hemisphere over time. Ipsilesional WMV reductions were larger in size and T score intensity compared to WMV reductions in the contralesional hemisphere. The pattern reflected brainstem and cerebellar input necessary for vestibular, somatosensory, and ocular motor processing. WMV reduction in the cerebral hemispheres was most prominent in ipsilesional thalamo-cortical connections to the insular-opercular region (PIVC), postcentral gyrus, premotor and cingulate cortex. (iii) In contrast to the extensive WMV loss, volumetric changes in GMV or cortical thickness as surrogate parameters for cortical atrophy were not detected (yet). Instead, a reduction of gyrification (GI), most prominently in the ipsilesional hemisphere, was observed as a result of the extensive WMV loss. This involved the PIVC (Ig, Ri, Op2-3), the mid- insula (PoI, MI) as well as the primary somatosensory cortex (area 2) and posterior parietal cortex (areas LIPv, VIP, 7PC). (iv) The functional connectivity analysis of the main affected thalamic nuclei in healthy individuals demonstrated brainstem / cerebellar-thalamocortical connections that reflect the WMV loss in the patient group and the functional specialization of these nuclei in ocular motor, vestibular and somatosensory processing.

### 4.1 Changes in cortical geometry and GMV

We did not detect any changes in cortical GMV and cortical thickness in the longitudinal analysis of our patient sample as markers of cortical atrophy. However, a reduction of gyrification was present in the cortical terminals of the extensive WMV reductions. Gyrification indices (GI) measure the sulcal compared to the outer cortical surface. (Luders et al., 2006) Therefore, lower values of GI reflect reduced cortical folding. This measure was introduced in neurodevelopmental and degenerative disorders, but, to our knowledge, has not been systematically studied in stroke patients in a longitudinal approach. (Caverzasi et al., 2019, Duret et al., 2018) It is our interpretation that the GI reduction here does not reflect gray matter volume loss, but is the consequence of cortical reshaping following widespread white matter atrophy after thalamic infarcts.

Contrary to previously reported brainstem or cerebellar infarcts, no gray matter volume changes were found. (Conrad et al., 2020, Conrad et al., 2021) This lack of gray matter changes, though somewhat surprising, might reflect the heterogenous interindividual projection pattern of the thalamus and the connections to a variety of areas in the cerebral cortex. Also, the multisensory integration centers that combine and exchange information from different sensory systems already at the brainstem level might be a reason and particularly difficult to evaluate in this regard. (Hwang et al., 2021) We are confident that longitudinal imaging with extensive permutation testing to study the remote cortical effects of a thalamic infarct was the most sensitive and reliable (against false positives) tool to detect cortical volumetric changes over time and provides sufficient statistical power do detect subtle changes over time. Our findings further suggest that cortical sensory plasticity after ischemic infarcts seems to rely on intact functioning of the thalamus as an integration center (i.e., GMV increases in response to a subcortical infarct). If the lesion affects the thalamus itself, cortical reorganization might be hindered, at least in the affected hemisphere.

### 4.2 Differences of central somatosensory and vestibular processing

We found remarkable differences in the course of recovery between somatosensory and vestibular symptoms. In contrast to other sensory modalities, vestibular and ocular motor signals are processed bilaterally and already integrated for both sides and with other sensory qualities at the level of the brainstem. (Brandt and Dieterich, 2017) Cortical vestibular and ocular motor processing is thus rarely unimodal. (Sommer and Wurtz, 2004, Dieterich and Brandt, 2015) Apart from early multisensory integration, the cortico-cortical ocular motor and vestibular network is highly redundant and therefore robust with a low network degree of vulnerability. (Raiser et al., 2020, Pierrot-Deseilligny et al., 2005) The results from this study further corroborate this pattern of robust and redundant central vestibular and ocular motor processing on the cortical level, resulting in a swift recovery of fundamental functions of balance and orientation. In contrast, injury to primary sensory areas cannot be compensated in a similar manner since early communication and substitution of the pathways from both sides of the body are missing.

### 4.3 Thalamic nuclei VPL and MD and vestibular, somatosensory, and ocular motor pathways

The thalamic subnucleus VPL conveys somatosensory input from lemniscal afferents to the cortex. (Jones, 2007) Spinothalamic fibers project to the ventral medial nucleus to the dorsal posterior insula and to the ventral caudal part of the mediodorsal nucleus to the anterior cingulate cortex. (Craig, 2002) Apart from connections to the somatosensory cortex, thalamocortical pathways from the VPL have been reported to the posterior insula and the posterior parietal cortex. (Impieri et al., 2018, Akbarian et al., 1992) WMV reductions in the cerebellar dentate nuclei region, the ML, the vestibular nuclei and hemispheric areas surrounding PIVC, somatosensory and premotor cortex could be detected in the longitudinal analysis and in the functional connectivity (rsfc) analysis using the lateral thalamic nuclei seed. Vestibular signals from the contralateral medial and bilateral superior vestibular nuclei reach the thalamus in lateral thalamic nuclei together with somatosensory input. (Wijesinghe et al., 2015, Lang et al., 1979) Lesion symptom mapping approaches (VLSM) have associated damage to these nuclei with vestibular graviceptive dysfunction such as tilts of the SVV. (Baier et al., 2016, Dieterich and Brandt, 1993) Furthermore, verticality perception is modulated in patients who receive deep brain stimulation in an area at the border of VL and VPL (nucleus ventralis intermedius of Hassler) which connects the dentate nucleus with the motor cortex. (Schaltenbrand, 1977, Baier et al., 2017)

Apart from its role in spinothalmocortical processing, the lateral portion of MD conveys saccade commands from the superior colliculi (SC) to the FEF, which act as a corollary discharge signal. (Sommer and Wurtz, 2004) The SC receives modulatory input from the vestibular system and cerebellum. (Leong et al., 2019, Wurtz and Goldberg, 1971) VLSM data demonstrated that lesions of MD are also associated with disturbed perception of verticality in stroke patients. (Baier et al., 2016) Recent imaging findings in macaques suggest rearrangements of thalamocortical connections between MD and the frontal eye fields after prefrontal lesions (PFC). (Adam et al., 2021) The WMV reduction in our patient sample and the results of the rsfc analysis likely reflect this SC-MD-premotor (FEF) connection.

Apart from ocular motor function, MD is involved in cognitive, emotional and attentional processes that rely on its connectivity with the PFC and limbic cortices as a hub in the default mode network (DMN). (Klein et al., 2010, Phillips et al., 2019) We refrain here from discussing the results of the current study in this context, as this was not the primary focus.

### 4.4 Limitations

We used a rigorous methodological approach with an intraindividual longitudinal design and extensive permutation testing to correct for type I errors in light of the group size. This has yielded reliable results in cohorts with similar sample sizes which is due to the longitudinal approach. (Van Ombergen et al., 2018) Additionally, similar results have been documented using combinations of surface analysis and tractography. (Chen et al., 2019)

The use of different scanners can lead to the introduction of a scanner bias in the results. Thus, we enrolled patients evenly balanced across the scanners and performed the longitudinal imaging (M0 and M6) on the same scanner in each patient. Furthermore, the T1-based tissue segmentations were harmonized during preprocessing via denoising and bias field correction. The data quality estimates as part of the CAT12 toolbox showed very good signal homogeneity for the different tissue types and all quality parameters were comparable.

While being heterogenous in terms of infarct side and the thalamic subnuclei affected, the sample was homogenous in the patients’ sensory qualities that we aimed to evaluate (i.e., vestibular and somatosensory processing in paramedian (MD) and lateral thalamic nuclei). (Baier et al., 2016, Craig, 2002, Dieterich and Brandt, 1993) To our knowledge, no significant differences between right- and left-sided thalamic processing have been reported for vestibular, ocular motor or somatosensory function and vestibular/ocular motor function is processed via both paramedian as well as lateral vestibular nuclei. (Baier et al., 2016, Kirsch et al., 2016) A reason for the homogenous sensory deficits in thalamic lesions is the early integration of vestibular and somatosensory information at brainstem level. While the issues mentioned above represent a potential limitation, we are confident that they did not bias our results in any way.

## 5 Conclusions

Volume loss after ischemic thalamic infarcts uncovers the white matter connections for sensory and ocular motor processing necessary for self-location in space and goal-directed actions. Over time, the WMV reduction seems to result in cortical deformation but not in gray matter volume loss.

Associated ocular motor and vestibular symptoms are compensated over time due to their redundant and intermingled connectivity and an early integration with other sensory modalities.

## CRediT authorship contribution statement

**Julian Conrad:** Conceptualization, Data curation, Formal analysis, Funding acquisition, Investigation, Methodology, Visualization, Writing – original draft. **Maximilian Habsa:** Investigation, Data curation, Writing – review & editing. **Ria M. Ruehla:** Investigation, Data curation, Writing – review & editing. **Rainer Bögle:** Investigation, Data curation, Methodology, Validation, Writing – review & editing. **Matthias Ertld:** Investigation, Data curation, Methodology, Validation, Writing – review & editing. **Valerie Kirsch:** Investigation, Data curation, Validation, Writing – review & editing. **Ozan E Eren:** Investigation, Data curation, Validation, Writing – review & editing. **Sandra Becker-Bensea:** Conceptualization, Data curation, Investigation, Methodology, Writing – review & editing. **Thomas Stephana:** Methodology, Writing – review & editing. **Frank A Wollenweber:** Conceptualization, Funding acquisition, Investigation, Methodology. **Marco Dueringe:** Conceptualization, Funding acquisition, Investigation, Methodology. **Peter zu Eulenburg:** Data curation, Formal analysis, Methodology, Visualization, Writing – original draft. **Marianne Dieterich:** Conceptualization, Funding acquisition, Investigation, Writing – original draft, Writing – review & editing.

## Declaration of Competing Interest

J. Conrad reports no disclosures relevant to the manuscript; R. M. Ruehl reports no disclosures relevant to the manuscript; M. Habs reports no disclosures relevant to the manuscript; R. Boegle reports no disclosures relevant to the manuscript; M. Ertl reports no disclosures relevant to the manuscript; V. Kirsch reports no disclosures relevant to the manuscript; O. E. Eren reports no disclosures relevant to the manuscript; S. Becker-Bense reports no disclosures relevant to the manuscript; T. Stephan reports no disclosures relevant to the manuscript; F. A. Wollenweber reports no disclosures relevant to the manuscript; M. Duering reports no disclosures relevant to the manuscript; P. zu Eulenburg reports no disclosures relevant to the manuscript; M. Dieterich reports no disclosures relevant to the manuscript.
